# Supplementary material for: “You don’t want anyone who hasn’t been through anything telling you what to do, because how do they know?”: Qualitative analysis of case managers in a hospital-based violence intervention program
Source: PLoS One. 2020 Jun 24;15(6):e0234608. doi: 10.1371/journal.pone.0234608 (PMC7313749; doi:10.1371/journal.pone.0234608)
Supplement: S1 Appendix — (DOCX) [file pone.0234608.s001.docx]

**Appendix A: Interview Guide**

1. What kind of community do you live in?
2. How did you first meet your case manager?

- *What was that experience like?*
- *How did you feel about him/her initially?*
- *Have those feelings changed or further developed? If so, how?*

1. How would you describe the relationship between you and your case manager now?
2. What do you like best about your case manager?

- *Tell me about a time your case manager came through for you.*

1. What do you wish you could change about your case manager?

- *Tell me about a time when your case manager wasn’t as helpful as you wished they could be.*

1. In what ways does your case manager understand your situation?

- *How were they able to gain that understanding?*
- *If they weren’t, why do you think that is the case?*

1. Looking back at your relationship with your case worker, is there anything that you would have done differently?
2. What advice do you have for people going through the Wraparound Project in terms of the relationship with their case managers?
3. In general, what attributes would make a good case manager for this program?

- *If we were hiring new people, what types of things would you consider?*

1. Have we missed something important? What else do you want to make sure we talk about?
2. Do you have any recommendations for improving the program?
