# Supplementary material for: “You don’t want anyone who hasn’t been through anything telling you what to do, because how do they know?”: Qualitative analysis of case managers in a hospital-based violence intervention program
Source: PLoS One. 2020 Jun 24;15(6):e0234608. doi: 10.1371/journal.pone.0234608 (PMC7313749; doi:10.1371/journal.pone.0234608)
Supplement: S2 Appendix — (DOCX) [file pone.0234608.s002.docx]

**Appendix B: Post Interview Contact Summary Sheet**

Contact Type:

Visit ________

Phone ______

Site:

Contact Date

Today’s Date:

Written by:

Interviewee:

1. What were the main issues or themes that struck you in this contact?
2. Summarize information you got (or failed to get) on each of the target questions.
3. What struck you as salient, interesting, illuminating, or important?
