# Supplementary material for: “You don’t want anyone who hasn’t been through anything telling you what to do, because how do they know?”: Qualitative analysis of case managers in a hospital-based violence intervention program
Source: PLoS One. 2020 Jun 24;15(6):e0234608. doi: 10.1371/journal.pone.0234608 (PMC7313749; doi:10.1371/journal.pone.0234608)
Supplement: Attachment — Submitted filename: Response to Reviewers 1.docx [file pone.0234608.s003.docx]

March 27^th^, 2020

Re: Revision of Manuscript Submission to PLOS One

The Academic Editor, PLOS One

Dear Janhavi Ajit Vaingankar,

Thank you to the Editorial team and reviewers for the opportunity to submit a revised version of our manuscript titled “You don’t want anyone who hasn’t been through anything telling you what to do, because how do they know?”: Qualitative Analysis of Case Managers in a Hospital-Based Violence Intervention Program for consideration for publication in PLOS One. We appreciate the thorough and insightful feedback from the reviewers. We have incorporated the suggested major and minor revisions and we feel that these edits have allowed us to significantly strengthen our manuscript.

We have addressed each comment and question received from the reviewers below. We thank you in advance for your consideration.

Sincerely,

Catherine Juillard, MD MPH

Associate Professor In-Residence

Department of Surgery, University of California, Los Angeles

Reviewer 1

The authors have conducted a thorough qualitative research study assessing the key points of strong, effective, therapeutic relationships between case managers and clients in the Hospital-based Violence Intervention Program setting. Although they used only one site to recruit patients, they included a sizeable number of interviewees which could feasibly reach theoretical saturation, or information power. While this is the first study of its kind, their results are not altogether surprising, and as presented, do not necessarily add much to the existing knowledge on this topic. I would recommend that the authors resubmit the manuscript after including more illustrative quotations from their interviews, and also attempting to generate conclusions which might contribute to the theoretical knowledge base on their topic. This might be done by extrapolating theoretical insights from their findings, or else, by relating their findings more specifically to existing literature. There is a rich amount of literature around therapeutic alliance, and it deserves reference within the context of this type of study. The authors note that their raw data is not available to the public; this is an incontrovertibly reasonable position to take given the social precarity of many of their research subjects. Overall it is an excellent study, but requires just a bit more work to make it an interesting and useful contribution to the field

1A. The authors should comment more specifically on what elements of information power were considered, and how the attributes of the interviewers were considered in this assessment.

RESPONSE: In conducting this project, we used the concept of information power to assess the sufficiency of our sample size, taking into account the study’s relatively narrow breadth, sample specificity, strategy the quality of the dialogue.^25^ Quality of the dialogue was particularly important given the interviewers were culturally discordant with the interviewed persons, though received training on trauma-informed interviewing and embedded themselves in the daily workings of the HVIP.” We have modified the methodology section to explicitly include these concepts.

1B. At what point in time were the clients in the sample seen by WAP? Were they actively engaged in case management, or had the relationship ceased, and for how long? Can you comment on how this temporal aspect may or may not have changed your results?

RESPONSE: Participants were interviewed after a six-month period of intensive case management had concluded, though some were still engaged in case management services to varying degrees. This involvement may have influenced participant responses, even though it was explicitly stated in the consent process that their participation or lack thereof in interviews would have no bearing on services provided and all conversations would be anonymized prior to being shared. We have updated the methodology section to include this timeline and have added its its potential effects to the limitations section.

1C. I am curious to know more about what factors can negatively impact the therapeutic relationship; Perhaps you can summarize this within a new/different theme, or at least address it more fully within the context of the themes you’ve already reported.

RESPONSE: Almost no participants were willing to expand on what, if any, factors had negatively impacted their relationships with their case managers – even when pressed. This may reflect the significant emotional potency of these relationships. This also may be due to the gatekeeper strategy employed to recruit participants – the case managers may have preferentially recruited clients with whom they had positive relationships, and clients may only have been willing to interview if they had a positive experience to share. Additionally, even though all clients had completed the intensive period of case management following the initial trauma, some were still engaged in case management services to varying degrees. This involvement may have influenced participant responses, even though it was explicitly stated that their participation or lack thereof in interviews would have no bearing on services provided and all conversations would be anonymized prior to being shared. This response has been included as a limitation under the discussion section.

1D. The length of the discussion is a bit cursory; the authors should attempt to make a contribution to theory, or at least to tie their results to more literature surrounding therapeutic relationships, case management, and trauma.

RESPONSE: Thank you for this important comment. The discussion section has been significantly revised to include comparisons to existing literature surrounding case management, trauma and therapeutic relationships.

1E. I would also add that, attempting to hire case managers with similar backgrounds can often present a challenge to HVIPs embedded in institutions that have strong biases against individuals who have interacted with the justice system, often times with restrictions against hiring individuals with prior felony convictions. Yet the need for cultural similarity is so paramount that hospitals should reconsider these restrictions.

RESPONSE: This is a very important point and is now referenced in the discussion section.

1F. Please expand on the potential impact of the language issue on your conclusions, and also on what the implications of the attributes of the interviewers are on your results.

RESPONSE: Though over half of WAP’s clients over the past decade have been English speaking, there may be needs of non-English speaking clients that were not captured in this study. Additionally, the language and gender of the interviewers were not concordant with those of some of the interviewees which also may have affected the depth of the interviews especially around sensitive topics such as interaction with law enforcement, mental health, immigration, and more. This important issue has been included as a limitation in the discussion section.

1G. I don’t find that these figures add much to your study. My preference would be to instead include a table with more illustrative comments from each of the themes.

RESPONSE: Although the decision was made to retain the figures in the manuscript based on other reviewer comments, we have added more illustrative comments from each of the themes.

Reviewer 2

I was pleased to review the manuscript, “You don’t want anyone who hasn’t been through anything telling you what to do, because how do they know?”: Qualitative Analysis of Case Managers in a Hospital-Based Violence Intervention Program” for publication in PLOS ONE. This research study was a qualitative analysis of the characteristics of case managers in hospital-based violence intervention programs (HVIPs) that maximize success when working with patients.

This study is particularly timely since HVIPs have expanded significantly over the past decade, including a push from the American College of Surgeons Committee on Trauma that tacitly nudges trauma centers to begin such programs. Despite this, there remains a large gap in knowledge, which this study helps to fill.

Overall, I found the content and results of the study to be a solid addition to the research base. Regarding the specifics on research methodology, I must defer to the advice of other reviewers as I have limited expertise in qualitative research/grounded theory methodology.

In general, I have minor revisions to suggest, which I will detail below:

2A. You report that participants “completed WAP’s period of intensive case management…” Can you simply quantify how long this period is?

RESPONSE: The section has been modified to now state the specific length of intensive case management for study participants.

2B. “Hand-written field notes were kept to track demographic information, contextual details, tone and key points in the interview.” Since the appendix provided doesn’t have any questions specific to tone, I would reword this. I’m sure tone may have been captured by the reviewers, but since it wasn’t specifically included, it’s uncertain if this was always reported.

RESPONSE: This section has been reworded and comments pertaining to tone have been removed.

2C. I have been debating in my head the validity of Figure 1 as presented. In the current presentation, it portrays the relationship between patient and case manager as progressing with time in a linear fashion from theme 1 to theme 6. Although this is a useful schema to think about the concept, my experience is that most patients progress in a non-linear fashion throughout time and the importance of different themes may come and go during the course of treatment. Unless the underlying data clearly shows that different themes were more important at different time periods, I would either remove this Figure or redesign it.

RESPONSE: Thank you for your comment. Language was added to emphasize that a relationship between patient and case manager is nonlinear; however, our interviews suggest that different themes are more important at different temporal points within the relationship which is the key point we hope to demonstrate by this figure.

2D. One point that struck me on multiple occasions was the use of the phrase “some participants” to describe themes. Since the phrase was used on many occasions, it left me wondering if all of those themes were brought up at roughly the same amount or if the themes were actually brought up with varying frequency (a small number of participants vs half vs most). Given the small sample size of this study, I do think that the use of the phrase is understandable, but if it is possible/appropriate to utilize words that describe the relative magnitude, it would be helpful for the reader. For example, if I were hiring for a case management position, I’d be very interested to know the intensity with which clients valued similar life experiences. Depending on that answer, it might change my hiring priorities.

RESPONSE: This point is well taken, and changes were made throughout the manuscript to reflect more accurately the number of clients describing these themes.

2E. The segment outlining Theme 4 could use additional details. As it reads now, it predominately discusses the risks of informal mentors and the challenges sometimes faced with formal mentors. The segment would benefit from additional details on why participants highlighted the importance of role models apart from the pitfalls of other information sources. A selected quote might be helpful.

RESPONSE: This segment has been significantly edited and a participant quote added to better outline the theme of case managers sitting at the intersection of formal and informal mentors.

2F. General thoughts on supplemental materials: Overall, I found the figures/graphs/appendices to be very helpful. With the exception of Figure 1 (discussed above), I thought the inclusion of the others were helpful in making concrete otherwise abstract concepts.

RESPONSE: We thank you for your comments on our use of figures, graphs and appendices and are pleased that these media helped make our project more concrete.

Reviewer 3

This is a well-designed, well-executed qualitative analysis of opinions of participants in an HVIP on preferable characteristics of HVIP case managers. These were: 1) understand and relate to client sociocultural context, 2) navigate in-hospital teachable moments to create connection with clients, 3) exhibit true compassion and care, 4) act as role models, 5) serve as portals of opportunity, and 6) engender mutual respect and pride.

Overall, this is a much needed patient-centered analysis on the qualities of an HVIP case manager that best serve clients. Many hospitals provide services to victims of violence (e.g. victim’s assistance offerings, police reporting at the bedside, information about support groups, medical coverage, and follow-up appointments for injuries). These services may be delivered by myriad staff (e.g. registration, technicians, clerical staff, nurses, social workers, physicians). However, delivering true trauma-informed care to victims of inner city violence requires someone to establish trust, have a shared understanding of life, show real empathy, and be dedicated and altruistic enough to continue their support after the hospital visit ends. This analysis not only provides a window into understanding the features of an HVIP case manager that are preferred by victims of violence, but also helps inform on how should be delivering services to these patients in the hospital setting.

Capitalizing on these teachable moments and forming a meaningful connection during the profound moment of injury can help prevent injury recidivism. However, too often we use ‘conventional’ outcomes such as re-injury or recidivism within the criminal justice system to measure success of HVIPs, failing to seek the perspective of those we serve. Although their cost-effectiveness has been shown, HVIPs alone cannot eliminate the social determinants of health that lead to violent injuries. However, HVIPs are an important part of the puzzle. What our clients feel matters and their connections expressed here with their case managers is positive and appreciative, providing hope for an all too often disenfranchised population. It is common sense that fearful patients would want “someone to talk to them on their level,” and that they would want someone who could put themselves in their shoes. Giving participants new opportunities for education, jobs, training, treatment, and safe physical spaces naturally builds participant resiliency and pride. With racial tensions often high in American cities, it is not surprising that case managers who share similar demographic features were often preferred by clients, but more important (particularly among female participants), was that case managers were open-minded and really cared. These features speak to how trust is gained. Participants want to be understood, just like every person. In fact, this study informs all of us on how to care for victims of violence, and I agree with the authors that “all individuals interacting with victims of violence in the healthcare system should be educated in trauma-informed care.”

Please note recommended edit below.

3A. Some participants did not prefer that their case manager share THEIR demographics or life experiences, but wanted THEM to be open to learning about THEIR life context without judgment. (Misplaced modifiers: unclear who these pronouns refer to--the participants or their case managers.) Recommend changing to: "Some participants did prefer that their case managers had similar demographics or life experiences. Rather, participants cared more that their case managers were open to learning about participants' lives without judgment."

RESPONSE: We thank you for your comment. We have updated this statement with your recommendation to clarify modifiers.
